# A study on the influence of online reviews of new products on consumers’ purchase decisions: An empirical study on JD.com

**DOI:** 10.3389/fpsyg.2022.983060

**Published:** 2022-09-09

**Authors:** Min Kang, Bing Sun, Tian Liang, Hong-Ying Mao

**Affiliations:** ^1^School of Economics and Management, Harbin Engineering University, Harbin, China; ^2^School of Foreign Studies, Harbin Engineering University, Harbin, China

**Keywords:** online reviews, purchase decision, text mining, LDA model, interaction effects

## Abstract

With the prevalence of the Internet and new media channels, consumer reviews have become one of the main determinants of Consumers’ purchasing decisions. This paper uses the Latent Dirichlet Allocation (LDA) model to identify the key factors that are of major concern to consumers, including design factors, laptop setup factors, logistics factors, after-sales factors, and user experience factors. And, we classify these factors into product quality factors and supporting service factors for new products. We then explore the relationship between online reviews and purchase decisions under these different factors, and also further explore the impact of interactions between online review metrics on purchase decisions. Our findings suggest that the impact of online reviews on consumer purchase decisions also varies considerably across different consumer focus factors. In addition, we find that the impact of the interaction between online review features is complex. In particular, consumers do not follow the positive guidance and make purchase decisions as we would expect when confronted with a large number of positive emotional polarity online reviews. Meanwhile, the interaction between negative emotional polarity and variance of online reviews had no significant effect on consumer purchase decisions. The variance of online reviews has a limited role in reducing consumer risk perceptions triggered by negative emotional polarity. Our study provides new evidence for the study of the impact of online reviews through text mining.

## Introduction

In the context of the globalized information age, enterprises are facing fierce market competition, and the need to rely on innovation to win a competitive advantage is becoming more and more urgent. Whether a new product, as a carrier of corporate innovation, can be accepted by the market and consumers determines the value of enterprise innovation (Nguyen and Chaudhuri, 2019). From the consumer’s point of view, any new or relatively new product that implements innovation or improvement to any part of the overall product concept and that brings a novel experience to people and meets their specific needs and interests can be called a new product (Cooper, 2019). Because of the “new” nature of new products, how effectively marketing them to consumers is undoubtedly a difficult task for companies. The stronger the consumer’s decision to purchase a new product, the more it can help companies achieve sustained sales growth while reducing operational risk and enhancing core competitiveness. However, according to statistics, although new products account for an average of 28% of a company’s sales profit, 41% of new products are still not successfully promoted (Cooper, 2012). According to the analysis, uncertainty is one of the main obstacles to the promotion of new products. Especially for high-tech products, customers generally find it difficult to understand the concept of new technology and new products and do not know what value and potential benefits the new products can bring to them (Wu et al., 2021). When consumers face a lot of uncertainty in a short period, it is difficult for them to make a quick purchase decision, and eventually, they may delay the purchase decision or even abandon the purchase of the new product. Therefore, early market word-of-mouth is crucial if companies want to bridge this gap in the marketplace. As stated by Robertson and Gatignon (1986), to reduce the functional risks, potential users often rely on the experience and opinions of other users to make purchase choices because they are unfamiliar with the new product and lack the knowledge framework to evaluate it.

Imagine that you are willing to buy a new laptop from a certain brand. Because of the lack of first-hand sensory information, the first thing most consumers do is to check and read product reviews to get a more realistic understanding of the features and functionality of a new product and decide whether to make a purchase decision. They trust the experience and opinions of consumers who have already purchased. In many cases, consumers make purchase decisions or cancel products from their shopping carts based on reviews. Such scenarios reflect the fact that with the rise of the Internet and e-commerce, online product reviews have become an important source of information for consumers to decide whether or not to purchase a product or which product to purchase. According to recent reports, 70% of consumers read online reviews of products before shopping, and 63% of consumers prefer to shop on websites with product reviews (MacDonald, 2018). Online reviews, as *a priori* user presentations, contain rich product information that can effectively reduce consumers’ perceived risk caused by information asymmetry during the shopping process. At the same time, because their sources are usually personal, online reviews are usually considered to have no commercial interest and are more trustworthy information (Erkan and Evans, 2016). Tang and Guo (2015) describe online reviews as “a gold mine of genuine customer reviews.”

There have been many studies that have explored the impact of online reviews on product sales in a valuable way (Sparks and Browning, 2011; Lau et al., 2016; Eslami and Ghasemaghaei, 2018; Eslami et al., 2018; Shankar et al., 2020; Byun et al., 2021), but research on online reviews for product attributes that are of greater interest to consumers is relatively rare. Such online reviews are more targeted and valuable, which is very important to both consumers and businesses. According to Moore (2015), online reviews that include product performance would be considered more helpful because it improves the consumer’s ability to evaluate the product. Especially for a new electronic products, such as laptops, consumers will evaluate the product in advance and build a knowledge model based on the information of product attributes (e.g., hard disk, processor, etc.). After pinpointing the product attributes that consumers care most about, online reviews containing information about these attributes are often what consumers value most and can directly influence their purchase decisions.

Therefore, we try to investigate the influence of online reviews of new products on consumers’ purchase decisions under different factors of consumers’ attention. Since online reviews often contain rich semantic information, including product features, user sentiment, delivery services, etc., how to extract information about users’ concerns from the massive unstructured data of online reviews and further mine the hot topics of consumers’ concerns becomes the basis of this study. As an unsupervised machine learning model for document topic document theme mining, the Latent Dirichlet Allocation (LDA) model proposed by Blei et al. (2003) can efficiently organize text information, transform unstructured text information into computable digital information, and identify hidden topic information in large-scale document sets. Based on the results of the LDA topic analysis of online reviews, this paper identifies the new product factors that consumers care most about. Then, combined with the indicators of online reviews, we use the two-stage least squares method (2SlS) to analyze the influence of various factors on consumers’ purchasing decisions and use the simultaneous equation model to deal with the endogeneity of the results.

The rest of the paper is organized as follows. First, we review relevant studies on the impact of online reviews on consumers’ purchase decisions. Next, we use the LDA model to identify the factors that consumers care most about based on online reviews. In the next section, we present the research hypotheses on the mechanisms of the role of online reviews under different factors. We then present the measurement and empirical models of the variables. To test the mechanism of action, we then empirically test the mechanism. Finally, the paper presents relevant theoretical contributions, managerial insights, research limitations, and future work.

## Literature review

The purchase decision involves a series of choices formed by the consumer before the purchase, once his/her willingness and needs are met, the purchase behavior is made (Hanaysha, 2018). As an important source of product information for consumers, word-of-mouth (WOM) communication among consumers can significantly influence the consumer attitudes and behaviors of WOM audiences; therefore, WOM has a significant impact on consumer purchase decisions (Richins, 1983). Especially for new products, consumers can only buy after perceiving (Plotkina and Munzel, 2016). WOM, as the main perception method, can help consumers understand product quality, decide whether to buy it and make judgments based on the product quality they experience after purchase; they can then guide this judgment with a certain probability to the consumer’s final purchase decision (Ozcan and Ramaswamy, 2004). In this process, WOM relies on consumers’ positive or negative emotional evaluations of products or services to exert powerful marketing power and business value.

The rise of new media channels over the past few years has provided fertile ground for electronic word-of-mouth (eWOM) communications (Cheung and Thadani, 2012). Consumers are no longer limited to face-to-face communication; they can spread their opinions and experiences of products through forums, shopping sites, consumer review sites, and other media, thus forming a wider and more influential eWOM. As the most popular and convenient eWOM communication method (Chatterjee, 2001), online reviews have gradually attracted the attention of scholars for their impact on consumer purchases. Especially in the Web 2.0 era, websites in different fields have emerged to provide consumers with more types of online reviews of products. Online reviews have become one of the main determinants influencing consumers’ purchase decisions (Weisstein et al., 2017).

Scholars have explored the impact of online reviews from a variety of perspectives. Early scholars have focused their discussions on films and books. For example, Lau et al. (2016) studied movie reviews and found that online movie reviews provided significant explanatory power for total box office revenues and weekly box office revenues and that most of this explanatory power came from the volume of WOM. It also confirms that valence, that is, the percentage of positive and negative reviews, does not affect sales. Forman et al. (2008) showed that the volume and length of online reviews, the number of reviews with a negative sentiment bias, and the level of information disclosure by reviewers affected book sales. Duan et al. (2008) developed a system of dynamic simultaneous equations to capture the relationship between online reviews and movie box office revenue. Their study found that while the valence of online reviews did not directly affect box office performance, higher valences were associated with greater volume of online reviews, which indirectly increased box office revenue. Then, several scholars have researched hotel reviews. Zhang et al. (2010) modeled the influence of online hotel reviews on consumer choice by applying the consideration set theory. They concluded that the affective tendency of reviews, consumers’ familiarity with the hotel, hotel hospitality, and reviewers’ professionalism all influence consumers’ stay decisions. Sparks and Browning (2011), Mauri and Minazzi (2013) concluded that online reviews have a significant impact on customers’ hotel purchase decisions and that there is a positive relationship between online review valence and hotel purchase decisions.

Meanwhile, some scholars have turned their attention to the field of electronics. Using a digital camera as an example, Floyd et al. (2014) suggested that online reviews have a greater impact on the sales elasticity of products with high engagement and that the valence and volume of reviews influence consumer purchases by affecting their perceptions. Chang et al. (2014) discussed the impact of online reviews around the variance of online reviews, which is “the degree to which reviewers agree on the product evaluation.” They classified reviews into factual and experiential reviews based on review variance and found that factual reviews with high variance for cell phones generated higher perceived usefulness and purchase intentions than factual reviews with low variance. Eslami et al. (2018) proposed a framework for online reviews best suited for new product sales based on a dataset of 1,500 sales of UGR digital cameras on Amazon. The framework consists of medium length, low review scores, and negative or neutral arguments. Li (2019) took laptops as an example, using the scenario simulation verification method to prove that certain positive emotions or negative emotions can bring consumers a higher perception of usefulness than uncertain emotions, and it is more conducive to consumers making shopping decisions. Then, based on data from Indian social networking sites, Shankar et al. (2020) proposed that online WOM is a triggering factor for consumers’ purchasing decisions, and combined with the ELM model to confirm that the quality, valence, volume, and variance of online reviews are beneficial to the localized dissemination of mobile banking. Through empirical research on the automotive industry, Byun et al. (2021) proposed multi-source review variance as a high-range inconsistency between product reviews reduces the diagnostics of product reviews, makes consumers feel less certain about new products; thus reduces the likelihood of consumer purchase.

Furthermore, with the gradual progress of research, in addition to the characteristics of online reviews, contents such as product attributes and service information contained in reviews have also gradually attracted the attention of scholars. This product-related information, as a product component, is considered to be an important factor influencing the consumer’s evaluation or choice of product (Kim and Kang, 2018). Several scholars have currently used multiple types of topic models to identify topics of consumer interest from consumer reviews. Lau et al. (2018) developed a big data architecture in combination with LDA topic models for product performance aspect level sentiment analysis and prediction of product sales. Then, Li et al. (2019) used a joint sentiment theme model to extract topics and related sentiments discussed more by consumers in review texts and found that two negative sentiment topics, interface and logistics and service, had no effect on product sales. However, positive emotional topics, including hedonic experiences and hardware, can affect product sales. Annisa et al. (2019) used the LDA model to extract eight topics from hotel reviews, namely rooms, facilities, breakfast, location of the hotel near the beach, tourist experience around the hotel, ambiance, hospitality, and bathrooms. They confirmed that these topics were frequently discussed as feedback between hotel management and tourists and were key factors influencing tourists’ choice of hotels. Based on consumer reviews of online refurbished smartphones, Nasiri and Shokouhyar (2021) identified topics in the reviews using the LDA model and found that the similarity of these products to brand new products and the lower price factor were the main reasons and motivations for consumer to purchase. And product features, including features related to satisfactory working, the appearance as the body and screen without scratches, and battery health, are the product features that concern consumers. Considering that consumer purchase decisions are influenced by emotions, Tian et al. (2021) identified the five most frequent topics in reviews based on Yelp restaurant review data by using semi-automatic content analysis methods: “food,” “service,” “Expense,” “Social,” and “Miscellaneous.” Then, they further analyzed the specific emotional tendencies of these five topics in conjunction with sentiment analysis. Wang et al. (2022) conducted an empirical study based on a review of charging infrastructure using a potential Dirichlet allocation topic model and a panel vector autoregressive model. They suggested that the critical reasons for negative consumer attitudes toward charging infrastructure are the inconvenience of charging, charging dilemmas, and the inability to install private charging posts. And the negative consumer attitude has a significant negative impact on the sales of new energy vehicles.

By summarizing the above studies, we find that scholars have studied the influence of various metrics of online reviews on consumers’ purchase decisions, but have not yet reached a consistent conclusion. For example, while most scholars believe that quantity and value positively influence consumers’ purchase decisions, some scholars find that these two characteristics do not influence purchase decisions, and some even argue that the quantity and value of online reviews hinder consumers’ purchase decisions. These contradictions in the literature suggested that there are still many black holes to continue to explore regarding the way online reviews operate and their limitations. We argue that, in addition to the differences in the research context and the research subjects themselves, current scholars have mostly conducted studies based on single metrics of online reviews; therefore, we attempt to provide more explanations for this inconsistency by further investigating the interactions among the metrics of online review and to shed light on the impact of online reviews on consumers’ purchase decisions. Then, current research on the impact of online reviews relies more on questionnaires and experimental analysis, while there are relatively few studies that deeply explore the content of consumer reviews containing rich product information (Zhang et al., 2018). Although some scholars have started to identify consumer concerns from online reviews by means of topic modeling, most of them directly explore the influence of the identified product topics on consumers’ purchase decisions. Only a few studies have combined the features of online reviews to investigate the impact on consumer purchase decisions, but they are only focused on sentiment analysis of online reviews. There is a lack of research that systematically combines the identified topic factors with multiple characteristics of online reviews to investigate the impact on consumer purchasing decisions. Therefore, we try to extract the features of product attributes that consumers care about by mining online review contents, and further investigate how online reviews under different new product attribute factors affect consumers’ shopping decisions. Finally, most of the existing studies on the impact of online reviews are based on existing products, and few explicitly discuss new products. In fact, due to the unknown characteristics of new products, the information from existing reviews can have a significant impact on subsequent consumers’ purchase decisions after they enter the market early. Therefore, it is important to explore the influence of online reviews based on the perspective of new products.

## Identification of new product factors that are of major concern to consumers in online review

### Research subjects

JD.com, an online shopping platform that enjoys a good reputation and popularity in China, was chosen as the source of data acquisition for this paper. This platform sells a large number of electronic products and can provide more complete online review data. In addition, we chose online reviews of new laptops as the object of our study. The object is considered appropriate because (1) the popularity of laptops in China is very high in recent years, and the online shopping volume is also large, (2) laptops are constantly innovating and new products are launched quickly; therefore, it is reasonable and feasible to use laptops as the research object to explore consumers’ purchasing decisions for new products, and (3) the diverse brands and product attributes of laptops help us identify the main factors that consumers pay attention to while considering different consumer groups.

**FIGURE 1:**
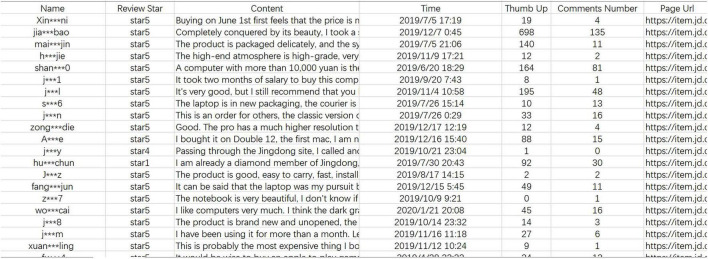
Crawl results of online review data for four popular laptops on the JD.com (partial).

This paper uses web crawler software developed based on Python to obtain online review data of four popular new laptops on JD.com, namely Apple MacBookPro, Lenovo Savior Y7000P, Dell Lynx 5580, and Asus SII generation. According to the rules of JD.com, we selected the four new models of laptops that hit the shelves in July−September 2019 and had the highest sales ranking by checking the new products and then sorting them by sales and combining them with the listing time shown on each laptop’s official website. Since the selected laptops are all products of the official flagship store of JD.com, which means that the launch time of the officially announced products is the same as the launch time of JD.com. And, online reviews are archived and indexed by release date. All reviews are dated according to when they were first posted, so we can continuously track weekly data on online reviews of newly listed laptops. Each piece of data we collected includes the consumer’s member name, member level, review star rating, review content, and review time, etc. (as shown in Figure 1). We set the data collection time to the earliest online review appearance time, and since the four types of laptops were listed and appeared for reviews at inconsistent times, we set the collection time from July 2019 to March 2020 to make the period of the reviews of the laptop with the latest review appearance time also reach 6 months. And we finally obtained 46,280 online consumer reviews data.

### New product factors that consumers are mainly concerned about based on the Latent Dirichlet Allocation model

#### Latent Dirichlet Allocation model

In this paper, the LDA model (Blei et al., 2003) is introduced to consumer reviews and identifies the factors that consumers care most about new products. In this model, the potential semantic structures in documents can be better discovered by an extended probabilistic implicit semantic analysis. This model has a three-layer Bayesian generation structure of “document-theme-word.” The probability of the occurrence of topic *k* in each document is multiplied by the probability of the occurrence of the word *w* under topic *k*, and then all topics are listed and summed to get the likelihood function of the entire document set M as follows:


(1) $$p=\left(w|\text{Θ},\text{Φ}\right)=\prod_{d=1}^{M}\prod_{i=1}^{{\text{N}}_{\text{d}}}p\left({\text{w}}_{\text{d},\text{i}}|{\text{θ}}_{d},\text{Φ}\right)$$

Among them, *w* represents the word of the whole document set; Θ represents the topic distribution of the whole document set *k*, that is Θ*_k_* ∼ Dir (α); and Φ represents the word distribution of the whole document set *d*, that is Φ*_d_* ∼ Dir (*β*). *N*_*d*_ is the total number of words of the *d*_*th*_ document, and w_di_ is the *i*_*th*_ word in of the *d*_*th*_ document.

Since Gibbs sampling algorithm has the advantages of being fast and efficient (Heinrich, 2005), it is used in this paper for parameter estimation about *α* and *β* in LDA models to transform high-dimensional word vectors into low-dimensional topic vectors. The critical for the LDA topic model to play a role in dimensionality reduction is the accurate setting of the number of potential topics for heterogeneous texts, but the LDA method itself does not generate the optimal number of topics. The number of topics is mostly determined by setting different values and comparing the best value after several verifications. Blei et al. (2003) proposed the using of Perplexity as a criterion for determining the number of topics, but it tends to lead to too much similarity between topics. Considering the generalization ability of the model and the effect of topic extraction, this paper uses the *Perplexity-Var* method (Welch, 2003; Li T. et al., 2022), which takes into account the perplexity and inter-topic similarity, to calculate the optimal number of topics. The method measures the structural stability of topics by their scatter and penalizes excessive overload of topics, minimizing the number of topics while ensuring maximum distinction between topics. The *Perplexity-Var* indicator is calculated by the following formula:


(2) $$\text{Perplexity}-\text{Var}\left(\text{D}\right)=\frac{\text{Perplexity}\left(\text{D}\right)}{\text{Var}\left(\text{T}\right)}$$

where *D* is the test set of the corpus, *Perplexity*(*D*) is the perplexity of the corpus set, and *Var* (*T*) is the topic variance of the corpus test set.

#### Identification of new product factors that consumers are mainly concerned about

After obtaining the data related to online reviews through Python, the data was pre-processed. Our data pre-processing consists of three tasks: (1) remove manually the garbled codes and duplicate reviews, and remove punctuation marks (e.g., !%$#&*?,/.;”\), (2) word segmentation and part-of-speech tagging. We apply the “Jieba” package in Python to accomplish this task. Since there is no interface to call lexicality directly in “Jieba,” we use jieba.posseg.cut for word separation and lexical annotation. Moreover, we add a manually summarized domain dictionary to ensure the quality of tokenization. Thus, ‘Power consumption’ will not be cut as “Power” and “consumption,” and (3) remove stop words. This step is an important task in data pre-processing. Stop words are usually a set of common words in any language (in this case Chinese) that should be removed from the document to focus on the important words. The construction of the stop word database is mainly to select the HIT stop word list (767) and the Baidu stop word list (1,395) for stop word de-duplication and integration, and the obtained stop word list is used as the initial stop word list. Then, the initial list of deactivated words was expanded according to the results of multiple topic analyses to add new high-frequency words that were not meaningful for the topic classification, such as: jingdong, review, computer, etc.

**FIGURE 2:**
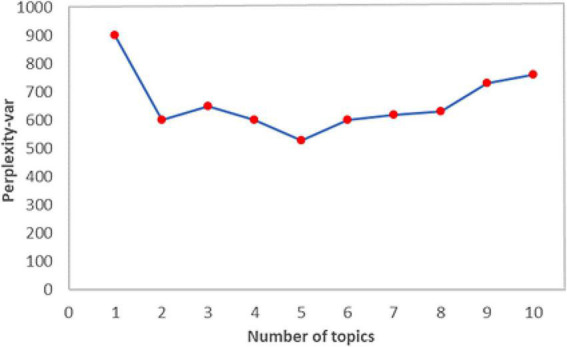
Perplexity-Var values of different topic numbers.

After preprocessing the text data of online reviews, we used the LDA model to model and analyze the text of the online reviews and then identified the topic words which reflect the factors that consumers are mainly concerned about and manually name the topic based on the logical relationship between these high probability words, to help us better understand the topic. In this paper, we use *Perplexity-Var* to evaluate the performance of LDA topic models and calculate the optimal number of topics. In the Gibbs sampling algorithm, the number of topics is selected with the parameter *α* = 50/*k* (*k* is the optimal number of topics), and the parameter *β* is set to 0.1 when the number of sampling iterations is100. The experimental process uses different numbers of topics for multiple clustering experiments. When the *Perplexity-Var* index is smallest, the corresponding LDA topic model is optimal. From the result of *Perplexity-Var* (Figure 2), we can see that the value reaches the minimum when the number of topics is 5, thus the optimal number of topics is determined to be 5 in the paper. From Table 1, we can see that the LDA topic model has a high degree of differentiation for each topic, and the clustering of topics is good, with related and similar words distributed in the same topic.

**TABLE 1:** Online review topic classification results.

	Topic1	Topic 2	Topic 3	Topic 4	Topic 5
Top 10 topic words	Package	Memory	Express delivery	After-sales	Sound quality
	Workmanship	Fitting	Speed	Attitude	Resolution
	Delicate	Screen	Attitude	JD Merchant Service	Cost performance
	Texture	Battery	Transport	Compensation	Sharpness
	Design	Application	Fast	Problem	Reaction
	Authentic	Gift	Service	Slow	Run
	Quality Color	Processor	Logistics	Experience	Power consumption
	Praise	Standby	Delivery	Return	Operating
	Level of	Game	Timely	thoughtful	Heat
	Appearance	Memory	Express delivery	After-sales	Sound quality
Topic name	Appearance design	Laptop setup	Logistics service	After-sales service	User experience

These five subject classifications are the main factors that consumers are mainly concerned about, including the appearance design factor, laptop setup factor, logistics service factor, after-sales service factor, and user experience factor. We further summarized the above five themes and found that appearance design, laptop setup, and user experience are all product quality factors; then logistics services and after-sales services belong to the supporting services of online shopping, which are an important way for e-commerce platforms to break through the shortcomings that consumers cannot directly feel the products (Hua and Jing, 2015). Therefore, we summarized these five themes as product quality factors and supporting service factors.

## Research hypothesis on the influence of online reviews on consumer purchase decisions under consumer concern factors

**FIGURE 3:**
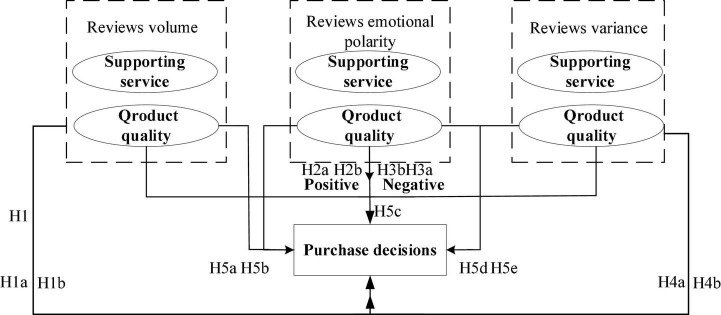
A conceptual model of the influence of online reviews on consumer purchase decisions.

Based on the identified main factors that consumers pay attention to, we will further explore the impact of online reviews on consumers’ purchase decisions under different factors. The EBM theoretical model states that as individuals become clear about their needs, they search for relevant product or service information to reduce the uncertainty of their purchase decisions (Engel and Roger, 1995). Therefore, the volume of online reviews affects consumers’ understanding of product information, which in turn affects their purchase decisions. Furthermore, prospect theory suggests that most people are more sensitive to losses than gains in the consumer decision-making process. Risk aversion and avoidance are important drivers of information seeking and decision-making for consumers (Campos-Vazquez and Cuilty, 2014). Thus, this paper also believes that the specific emotional polarity conveyed by online reviews and the review variance that reflect the reviewer group preference divergence are both key metrics that affect consumers’ shopping decisions. Note that the current star distribution of reviews is a J-shaped distribution (rather than a more uniform U-shaped distribution), suggesting that the validity of online product reviews is overwhelming, with 5-star ratings being the dominant category, some 1-star ratings, and few ratings in between (Ullah et al., 2015). For more precise identification of consumer emotional polarity, we will use the machine learning-based Baidu Cloud API sentiment analysis tool to accurately classify each review’s emotional polarity. Therefore, in this paper, we construct a theoretical model of online review volume, online review emotional polarity (positive and negative), and online review variance on consumer purchase decisions (shown in Figure 3).

### Online review volume and consumer purchase decision under different factors

The volume of online reviews not only provides a direct measure of the overall size of online reviews but also reflects the popularity of the product among consumers (Zhang et al., 2013). The volume of online reviews of a product posted by consumers can have a profound impact on the purchase decisions of potential consumers. On the one hand, in the early stage of a new product launch, the more online reviews, the more awareness effect of consumers on the new product will be improved (Zhou et al., 2019). Moreover, consumers can obtain relevant product information promptly, reduce purchase uncertainty, and make purchase decisions as soon as possible. On the other hand, when there are a large number of online reviews of a new product, it means that there are many recipients and buyers of the new product and that the product is generally considered to be a “quality product” (Yang et al., 2016). For example, on many shopping sites such as Amazon, consumers can retrieve new products based on the number of reviews, which directly reflects the popularity of the product (Kordrostami et al., 2020). Under the influence of the “Popularity effect,” it is more likely to generate subsequent sales (Erkan and Evans, 2016).

Therefore, this paper proposes this hypothesis.

H1: The volume of online reviews of new products has a positive effect on consumers’ purchase decisions.

A higher number of online reviews about product quality factors of new products can provide more accurate and detailed information about product performance and consumer experience, and reduce consumer perceptions of shopping risks (Chien et al., 2016), which in turn enhance consumers’ purchase decisions. Also, the more online reviews that provide feedback on product functions and features, the more potential consumers can get the experience of the product with less cost and transaction risk, which facilitates potential consumers to generate purchase decisions. At the same time, most transactions on the Internet rely on the use of logistical channels to deliver products (Xu et al., 2019). The more online reviews about logistics and after-sales, the more information consumers can obtain about timeliness, quality of delivery, communication during product delivery and distribution, and basic warranties to correct product defects or failures (Rahman and Chattopadhyay, 2015). This information can enhance consumers’ perception of the reliability of a new product and can motivate their purchase decisions. Existing research suggested that warranty time and after-sales can support and enhance the use of relatively durable devices that have been purchased, and are therefore important factors influencing consumer purchases of new PCs (Nochai and Nochai, 2011).

Therefore, this paper proposes this set of hypotheses.

H1a: The volume of online reviews of the product quality factor of a new product has a positive impact on consumer purchase decisions.

H1b: The volume of online reviews of the supporting service factor of a new product has a positive impact on consumer purchase decisions.

### Emotional polarity of online reviews and consumer purchase decisions under different factors

Online reviews with clear emotional polarity reflect the positive and negative attitudes of purchasing consumers toward product attributes after experiencing a new product (Liu and Karahanna, 2017). The emotional infection theory of psychology proposes that people automatically imitate and merge the verbal and non-verbal messages of others and consciously incorporate the emotions of the emotion transmitter (Elfenbein, 2014). Therefore, potential consumers tend to follow the attitudes conveyed in the reviews for their shopping choices (Filieri et al., 2018).

When reviews of the laptop’s design, system, memory, and accessories convey positive emotional polarity, this indicates that the new product’s functions and features meet consumers’ psychological expectations and subjective perceptions. Such reviews can enhance the pleasure of potential consumers, trigger their senses, and increase their purchase decisions to a great extent (Triantafillidou and Siomkos, 2014). Scholars also point out that receiving goods quickly and accurately and receiving good after-sales service are important requirements for current consumers in the online shopping process (Lyu and Choi, 2020). These requirements are directly related to consumers’ perceived service quality. Therefore, when reviews convey positive emotional polarity about the supporting services, they can enhance potential consumers’ purchase confidence and promote purchase decisions. In contrast, when online reviews contain negative emotional polarities, consumers are strongly influenced by negative emotions due to their own risk-averse mentality, and have a stop-loss mentality in time. Thus, it is not conducive to consumers’ purchase decisions.

Therefore, this paper proposes another set of hypotheses.

H2a: Positive emotional polarity of online reviews regarding the product quality factor of the new product has a positive impact on consumer purchase decisions.

H2b: Positive emotional polarity of online reviews regarding the supporting service factor of the new product has a positive impact on consumers’ purchase decisions.

H3a: Negative emotional polarity of online reviews regarding the product quality factor of the new product has a negative impact on consumers’ purchase decisions.

H3b: Negative emotional polarity of online reviews regarding the supporting service factor of the new product has a negative impact on consumers’ purchase decisions.

### Variance of online reviews and consumer purchase decisions under different factors

Online review variance, which mainly measures disagreement or heterogeneity among customers, is the main measure of the validity of online reviews (Kostyra et al., 2016). Reviewers usually hold different opinions about the same product (Sun, 2011). In research related to cognitive dissonance theory, Sidnam-Mauch and Bighash (2021) found that individuals feel psychological ambivalence and anxiety when faced with information that has variance and tends to reduce the variance by changing their attitudes or changing their behavior. The current laptop has evolved from a pure productivity tool to a powerful and creative user environment (Hoyle et al., 2013). When faced with online reviews with high variance, consumers will be in a dilemma of not knowing how to judge the performance of the product. Consumers subconsciously believe that the product is not unanimously approved, and thus they have doubts about the product and are more uncertain about the expected outcome of the purchase, which increases their risk perception of the product. At this point, consumers will not make a purchase choice (Petersen and Kumar, 2015). Conversely, when online reviews have low variance, reviews can provide consumers with specific and practical reliability discussions about laptop, thereby enhancing consumers’ purchase interest.

In the online environment, customers’ shopping behavior has a certain delay, and goods need to go through a third party to reach them, especially for high-value electronic products, consumers have high requirements for the timeliness and reliability of product logistics and after-sales services (Lin et al., 2003). When the variance of online reviews is high, it means that the logistics and after-sales services such as delivery speed, response time, repair speed, and service attitude of the products have gained a strong controversial discussion. At this point, potential consumers believe that the product’s shipping quality and after-sales service are unstable (Mudambi and Schuff, 2010), and their perceived emotions will fluctuate even more. Consumers will not be able to determine the logistics and delivery service as well as the after-sales guarantee, which will prevent them from making purchase decisions.

Therefore, this paper proposes an alternative set of hypotheses.

H4a: The variance of online reviews regarding the product quality factor of the new product has a negative impact on consumer purchase decisions.

H4b: The variance of online reviews regarding the supporting service factor of the new product has a negative impact on consumers’ purchase decisions.

### The interaction between online review metrics and consumer purchase decisions

Consumers are induced to buy new products when a large number of online reviews about design, laptop setup, logistics and after-sales, user experience, etc. have positive emotional polarity. This is mainly because when the affective polarity of online reviews is positive and the volume of reviews is high, a large amount of positive review information about these factors that consumers care about is passed on to consumers; thus increasing potential consumers’ confidence in the quality or performance of the new product (Huyen and Costello, 2017) and helping them to make a quick purchase decision. On the contrary, when the sentiment polarity of online reviews under different factors is negative and the volume of reviews is high, it will prevent consumers from purchasing new products. Thus, it can be seen that the interaction between online review volume and emotional polarity under different factors also affects consumers’ purchase decisions.

Accordingly, the following hypotheses are proposed in this paper.

H5a: The pairwise interaction between the volume and the positive emotional polarity of online reviews under the product quality factor has a positive effect on the consumer purchase decision.

H5b: The pairwise interaction between the volume and the negative emotional polarity of online reviews under the product quality factor has a negative impact on consumer purchase decisions.

At the same time, when online review variance is high, more online reviews make it more difficult for consumers to extract relevant and useful information from reviews about the design, settings, and user experience of a new laptop. Consumers will have limited information processing capabilities and will be unable to distinguish the authenticity and validity of online review information (Shao et al., 2018). According to dual-process theory, consumers automatically use heuristic processing mechanisms to reduce cognitive overload. Heuristic processing requires low levels of cognitive engagement and allows people to make quick decisions through intuition. Therefore, it is expected that people will ignore the negative aspects of the presented information and will be attracted only by the interesting properties of the variance itself. We argue that when the volume of online reviews is large, the facilitative effect of online reviews on consumers’ purchase decisions will significantly outweigh the negative effects of online review variance (Li H. et al., 2022). Thus, the interaction between online review volume and variance of a new product has a facilitative effect on consumer purchase decisions. As proposed by Khare et al. (2011), higher online review volume is more diagnostic and can absorb negative consumer perceptions of the product relative to low online review volume.

Therefore, this paper proposes the following hypothesis.

H5c: The interaction between the variance and the volume of online reviews under the product quality factor has a positive effect on consumer purchase decisions.

In addition, the higher the variance of online reviews regarding design, laptop setup, logistics and after-sales, and user experience, the greater the bias in consumer reviews of the new product. Clemons et al. (2006) suggested that when products are differentiated, some people will find a perfect match with their preferences among different products and give good ratings, while others with different preferences will provide the opposite ratings. Uncertainty, represented by high variance, favors products with low ratings and disadvantages products with high ratings (Kostyra et al., 2016). When consumers are confronted with online reviews that have a positive affective bias, higher review variance can reduce potential consumer purchase decisions because it can cause potential consumers to question the positive reviews and they may perceive that some consumers seem to be dissatisfied with the new product. Therefore, consumer confidence in purchasing the new product would be affected. And negative emotional polarity and lower online review variance will increase consumers’ purchase decisions for new products. Accordingly, this paper proposes the following hypothesis.

H5d: The interaction between positive emotional polarity and online review variance under the product quality factor has a negative effect on consumers’ purchase decisions.

H5e: The interaction between negative emotional polarity and online review variance under the product quality factor has a positive effect on consumers’ purchase decisions.

## Research methodology

### Measurement of the variables

The volume index of online reviews is measured by the number of online reviews under different factors. By filtering the crawled data of 46,280 online reviews of laptops, 45,801 reviews were obtained in this paper. Among them, the number of reviews under the product quality factor and the supporting service factor are 28,282 and 17,519, respectively.

**FIGURE 4:**
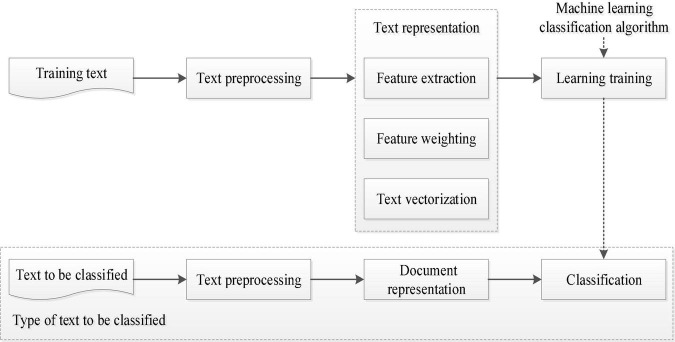
Emotional polarity analysis process based on machine learning.

The emotional polarity analysis of online reviews mainly identifies the text semantics of the online reviews of a product to obtain the emotional polarity of potential consumers toward the product, that is, to determine the degree of praise and criticism of the text content of the online review (Mohammad, 2016). The essence of the emotion polarity recognition method based on machine learning is to use the trained classifier to identify the emotional polarity of the analyzed reviews, that is, to classify the relevant emotional expression objects into two categories of positive and negative emotions. In this paper, the specific process of emotional polarity analysis of useful online reviews under different factors is shown in Figure 4. The first part of the figure is the preprocessing process of the text, which processes the online review text according to the needs, such as word segmentation and so on. The second part is the document representation. Select the appropriate feature to represent the text, express the importance of the feature by weighting, and then vectorize the text so that it can be processed by a computer. The third part is the training learning process, which uses the classification model to constantly learn and train the manually labeled samples to establish a classifier suitable for this kind of text. The last part is to classify the text, that is, to classify the online reviews of various factors according to the emotional polarity. This paper adopts the method of the Baidu Cloud API emotional analysis tool based on machine learning to realize the above process and obtains the emotional polarity analysis results of online reviews with different factors (as seen in Table 2). This lays the foundation for the later use of regression models to analyze the influence of the emotional polarity under different factors on the consumers’ purchase decision.

**TABLE 2:** Analysis of the emotional polarity of online reviews under different factors.

Factor	2 (Positive)	1 (Negative)
Products quality factors	18343 (57%)	5149 (16%)
Supporting services factors	11099 (63%)	3347 (19%)

Most existing studies use the variance of overall consumer ratings to measure the variance of online reviews. Therefore, we refer to the study of Clemons et al. (2006) and use the dispersion of the WOM distribution to calculate the heterogeneity of consumer reviews of each new laptop. Specifically, each review on JD.com has a numerical rating on a scale of 1 to 5 stars, where 1 star corresponds to the least satisfied and 5 stars corresponds to the most satisfied with the product. We used the variance of the overall consumer-rated reviews as a variable to measure the magnitude of review heterogeneity, with a larger variance indicating higher heterogeneity.

For the measurement of purchase decisions, this paper uses inverted sales ranking as a measure of new product sales. Since JD.com does not disclose the specific sales data of its products, similar to other e-commerce sites, this paper cannot directly obtain the sales data of products purchased by consumers. JD.com only provides weekly sales rankings of products, which indirectly reflect product sales. For most products, the relationship between sales rank and sales can be represented by the Pareto distribution (also known as the 80/20 rule), which implies that the relationship between *lnsale* and *ln*(*salesrank*) is roughly linear (Chevalier and Goolsbee, 2003). The only difference between the linear models using *lnsale* and using *ln*(*salesrank*) is the way the estimated coefficients and standard errors are measured, and these differences do not change the sign and significance of the coefficients, as verified by Sun (2011), Kaushik et al. (2018), and the equation uses *ln*1/*salesrank* can make the sign of the coefficients easier to interpret. Therefore, the natural logarithm of the inverse of the weekly computer sales ranking of the four laptops is also used to replace their sales values in this paper.

### Empirical model specification

(1) Construction of the benchmark model:

To test the influence of online reviews of new products on consumers’ purchase decisions, we developed a benchmark test model of online review metrics that included both product quality factors and supporting service factors. And refer to the research of Duan et al. (2008), Ryoo et al. (2021), we also include a lagged dependent variable in the model to capture the effects of all factors that may have influenced purchases in the past but were not included in the model. In addition, to reduce the effect of heteroskedasticity, the logarithmic form of all-time series variables is taken in this paper.


(3) $$lnPurchase_{i,t}={\text{θ}}_{t}+{\text{α}}_{1}lnPurchase_{i,t-1}+{\text{α}}_{2}\ln Volume_{i,t}$$

$$\;+{\text{α}}_{3}\ln QV_{i,t}+{\text{α}}_{4}\ln SV_{i,t}+{\text{α}}_{5}\ln QP_{i,t}$$

$$\;+{\text{α}}_{6}\ln SP_{i,t}+{\text{α}}_{7}\ln QN_{i,t}+{\text{α}}_{8}\ln SN_{i,t}$$

$$+{\text{α}}_{9}\ln QA_{i,t}+{\text{α}}_{10}\ln SA_{i,t}$$

$$\;+\sum {\text{ρ}}^{*}Control_{i,t,}+{\text{μ}}_{i}+{\text{ε}}_{i,t}$$

In the above formula, *i* = 1, 2 …… *N* represents each newly listed laptop selected in this paper, and *t* represents time (in weeks). *Purchase*_*i*,*t*_ represents the consumer purchase decision of product *i* in the market in week *t*, and its one-week lagged variable is represented by *Purchase*_*i,t–1*_. Correspondingly, ln *QV*_*i*,*t*_, ln *QP*_*i*,*t*_, ln *QN*_*i*,*t*_ and ln *QA*_*i*,*t*_ represent the cumulative volume, positive emotional polarity, negative emotional polarity, and variance of product quality factors in week *t*, respectively. In the same way, ln *SV*_*i*,*t*_, ln *SP*_*i*,*t*_, ln *SN*_*i*,*t*_ and ln *SA*_*i*,*t*_ represent the cumulative volume, positive emotional polarity, negative emotional polarity, and variance of supporting services factors in week *t*, respectively.

Meanwhile, ∑ ρ*Control_*i*, *t*_ is the set of explanatory variables, mainly including the price of the product (*lnPrice*_*i, t*_), and timeliness (*lnTimeliness*_*i,t*_), the length of online review (*lnlenth*_*i,t*_), in addition, there is a dummy variable, namely sales promotion (*Spromotion*_*i,  t*_). *lnPrice*_*i*,*t*_ represents the actual sales price of product *i* on JD.com in week *t* (we use the average price of this week to calculate). *lnTimeliness*_*i,t*_ denotes the time difference of new product reviews, that is, the time difference from the launch time of a new product to the time of making a review (Luo et al., 2021). *lnLenth*_*i,t*_ denotes the online reviews length, which is calculated by the total number of review characters. *Spromotion*_*i,  t*_ indicates whether there is a large-scale promotion of product *i* in week t (according to the event calendar displayed on the official website of JD.com). It is defined here that *Spromotion*_*i*, *t*_ = 1 when there is a promotion, and *Spromotion*_*i*, *t*_ = 0 when there is no promotion.

In addition, θ_*t*_ is the intercept terms of the purchase decision and represent the total time difference of each newly launched laptop. For equation, combined with fixed effects, μ_*i*_ is used to capture other random factors that affect the purchase of laptops in addition to price, sales promotion, timeliness, and length of online review such as the marketing investment of each new product. The fixed effect captures the unobservable heterogeneity of each laptop product that does not change over time. Based on this, the model settings also control the unobserved differences between different laptops. In addition, fixed effects also allow the residual term ε_*i*,*t*_ to be related to other explanatory variables, which makes model estimation more flexible and robust.

This paper first presents the descriptive statistical analysis of each variable and analyzes the correlation of each variable. From the correlation matrix between variables in Table 3, it can be seen that the absence of multicollinearity. And, there is a strong correlation between the volume, positive emotion polarity, negative emotion polarity, variance, and purchase decision; especially the volume of online reviews (ln *Volume*) and the negative emotional polarity of product quality factors (ln *QN*) and purchase decisions are even stronger.

**TABLE 3:** Summary statistics analysis.

Variable	Mean	SD													
*lnPurchase*	6.18	2.05	1												
ln *Volume*	16.12	3.59	0.61	1											
ln *QV*	35.79	9.15	0.41	0.23	1										
ln *SV*	21.11	4.79	0.17	0.18	0.06	1									
ln *QP*	−1.15	1.08	0.28	0.15	0.08	0.15	1								
ln *SP*	−0.49	0.45	0.11	0.15	0.13	0.17	0.12	1							
ln *QN*	−0.19	0.14	−0.43	−0.23	0.05	0.02	0.06	0.05	1						
*lnSN*	−0.26	0.15	−0.29	0.31	0.11	0.19	0.05	0.02	0.13	1					
ln *QA*	0.73	0.41	0.25	0.05	0.22	0.25	−0.31	0.15	0.01	0.02	1				
ln *SA*	0.64	0.23	0.33	0.03	0.04	0.12	0.02	0.17	0.21	0.11	0.04	1			
*lnPrice*	18.42	4.46	−0.15	−0.10	−0.02	−0.02	0.04	0.43	0.10	0.00	0.21	0.34	1		
*ln*timeliness	5.49	3.67	0.18	0.07	0.12	0.11	0.11	0.17	0.37	0.08	0.14	0.14	0.14	1	
*lnlenth*	3.87	0.53	0.25	0.14	0.14	0.05	0.13	0.07	0.00	0.10	0.04	0.01	0.07	0.07	1

(2) Construction of interaction model:

Considering the interaction effects among the metrics of online reviews of new products can also have an impact on new consumers’ purchase decisions. Therefore, we include the interaction terms of online review metrics in the model:


(4) $$lnPurchase_{i,t}={\text{θ}}_{t}+{\text{α}}_{1}lnPurchase_{i,t-1}+{\text{α}}_{2}\ln Volume_{i,t}$$

$$\;+{\text{α}}_{3}\ln QV_{i,t}+{\text{α}}_{4}\ln SV_{i,t}+{\text{α}}_{5}\ln QP_{i,t}$$

$$\;+{\text{α}}_{6}\ln SP_{i,t}+{\text{α}}_{7}\ln QN_{i,t}+{\text{α}}_{8}\ln SN_{i,t}$$

$$\;+{\text{α}}_{9}\ln QA_{i,t}+{\text{α}}_{10}\ln SA_{i,t}+{\text{β}}_{1}\ln QV_{i,t}$$

$$\;*\ln QP_{i,t}+{\text{β}}_{2}\ln QV_{i,t}*\ln QN_{i,t}+{\text{β}}_{3}\ln QA_{i,t}$$

$$\;*\ln QV_{i,t}+{\text{β}}_{4}\ln QP_{i,t}*\ln QA_{i,t}+{\text{β}}_{5}\ln QN_{i,t}$$

                          *lnQAi,t+∑ρ*Controli,t+μi+εi,t

In the model, we add the interaction between the characteristic indexes of online reviews about product quality factors, namely, ln QV_i,t_* ln *QP_i,t_* and ln QV_i,t_* ln *QN_i,t_* represent the interaction between the volume of online reviews and positive emotional polarity, and the volume of online reviews and negative emotional polarity, respectively. ln QA_i,t_* ln *QV_i,t_* represents the interaction between the variance and volume of online reviews. ln QP_i,t_* ln *QA_i,t_* and lnQN_i,t_* ln *QA_i,t_* represent the interactions between the positive emotional polarity of online reviews and the variance, and the negative emotional polarity and variance of online reviews, respectively.

## Results and discussion

### Analysis of baseline regression results

During the above empirical tests, although we have included control variables in the regression models to minimize endogeneity problems caused by omitted variables as well as estimation bias, the core explanatory variable, the volume of online reviews, may still be related to other uncontrolled third-party factors that simultaneously influence consumer purchase decisions, biasing the estimated coefficients. Thus, simply estimating the equation using ordinary least squares (OLS) may lead to variance in the estimation results. Thus, referring to the study of Oberholzer-Gee and Strumpf (2007), Lee et al. (2015), we use the more efficient the two-stage squares estimation (2SLS) to estimate the Eq. 2 (Model 2-Model 3); we then list the OLS estimation results for comparison as well (Model 1).

Meanwhile, we introduced instrumental variables (IV) to further address the endogeneity problem caused by omitted variables and thus ensure the accuracy and stability of the results. We use the total number of useful votes for a one-week (one cycle) lag of all reviews for a new product, that is, the total useful votes for new product *i* in week *t − 1*, as IV for the volume of online reviews in week *t*. Helpful votes are voted by consumers who have read the reviews and are therefore positively correlated with the total number of online reviews, the endogenous variable. Also, the number of helpful votes is a longer-term and slowly moving total (Sande and Ghosh, 2018). Also, to further ensure independence from new product purchases, we used a 1-week lag total number of helper votes. In addition, we also tested the validity of the instrumental variables. According to the results of the first stage estimation of 2SLS, namely Model 2, the Kleibergen-Paaprk LM statistic is 14.572, corresponding to a *p*-Value less than 0.01, indicating that there is no under-identification of instrumental variables. Similarly, the Kleibergen-Paaprk Wald F-statistic is 23.583, which is greater than the critical value at the 10% significance level, indicating that there is no weak instrumental variable identification problem. Therefore, the instrumental variables selected in this paper are more valid. The regression results of the baseline model are shown in Table 4.

**TABLE 4:** Results of the influence of online reviews on consumers’ purchase decisions.

	Model 1	Model 2	Model 3

	**OLS**	**2SLS (The first stage)**	**2SLS (The second stage)**

	**Purchase decision**	**Volume**	**Purchase decision**
*lnPurchase*_*i,t–1*_	0.648*** (0.107)	0.563** (0.219)	0.673*** (0.120)
ln *Volume*_*i, t*_	0.611*** (0.107)	–	0.662*** (0.107)
ln *QV*_*i*,*t*_	0.572** (0.189)	0.113* (0.052)	0.596** (0.139)
ln *SV*_*i*,*t*_	0.586* (0.239)	0.075 (1.650)	0.593** (0.190)
ln *QP*_*i*,*t*_	0.235* (0.110)	0.051 (0.45)	0.232* (0.113)
ln *SP*_*i*,*t*_	0.229* (0.078)	0.205* (0.091)	0.093* (0.184)
ln *QN*_*i*,*t*_	−0.877 (0.349)**	−0.521 (0.179)**	−1.017** (0.345)
ln *SN*_*i*,*t*_	−0.621** (0.238)	0.193 (1.201)	−0.659** (0.362)
ln *QA*_*i*,*t*_	−0.737* (0.312)	0.245 (0.790)	−0.735 (0.292)
ln *SA*_*i*,*t*_	−0.533* (0.264)	0.397 (1.17)	−0.547* (0.263)
*ln*helpfulness_*i*,*t*−1_	–	0.447*** (0.137)	–
*ln*Pric*e*_*i*,*t*_	−0.314** (0.154)	0.376*(1.913)	−0.261** (0.103)
*ln*Timeliness_*i, t*_	0.409* (0.200)	0.028 (0.513)	0.121 (0.139)
*lnLenth*_*i,t*_	0.336* (0.170)	0.016 (0.219)	0.219* (0.106)
*Spromotion*_*i,t*_	0.327** (0.147)	−0.360 (0.437)	0.349* (0.167)
Cons-	3.688* (1.563)		4.018** (1.398)
Kleibergen-Paaprk LM Statistics	–	14.572 [0.00]	–
Kleibergen-Paaprk Wald F Statistics	–	23.583{19.93}	–
N	45801	45801	45801
R^2^	0.732	0.861	0.747

*p < 0.05, **p < 0.01, ***p < 0.001; values in parentheses are robust standard errors; values in middle brackets are p-values of statistical tests; values in large brackets are critical values for the 10% level of the stock yogo weak instrumental variable test.

As can be seen in Table 4, lagged shopping decisions (*lnPurchase*_*i,t*–1_) positively influence current purchase decisions (α_1_ = 0.673, *p* < 0.001) . This suggests that there is indeed a persistent feature of consumer purchase decisions and that prior shopping decisions positively influence current ones.

(1) Influence of online review volume on consumers’ purchase decisions under different factors:

From the 2SLS estimation results in Table 4 (Model 3), it can be seen that the volume of product reviews (ln Volume_*i*,*t*_) is significantly and positively correlated with consumers’ purchase decisions (α_2_ = 0.662, *p* < 0.001), and H1 is verified. Meanwhile, the volume of online reviews for the product quality factor (ln *QV*_*i*,*t*_) and for the supporting service factor (ln *SV*_*i*,*t*_) also have a significant positive effect on consumers’ decision to purchase new products, thus supporting hypotheses H1a and H1b. Moreover, the regression coefficient of the product quality factor (α_3_ = 0.596, *p* < 0.01) is greater than that of the supporting service factor (α_4_ = 0.593, *p* < 0.01). This is mainly because, in this information age, the quality of hardware such as the screen, graphics card, and hard disk of laptops is directly related to the working, learning, and entertainment functions of laptops; therefore, product quality has been a common concern among consumers. Consumers will try to read a lot of reviews about the quality of laptops to further understand the real situation of laptops, and they will expect various performance requirements to match each feature of the product (Lyu and Choi, 2020).

(2) Influence of positive emotional polarity of online reviews on consumers’ purchase decisions under different factors:

As can be seen from Model 3 in Table 4, the positive polarity of the product quality factor (ln *QP*_*i*,*t*_) and the supporting service factor (ln *SP*_*i*,*t*_) have a positive effect on consumer purchase decisions (α_5_ = 0.232, *p* < 0.05; α_6_ = 0.093, *p* < 0.05), supporting H2a and H2b. However, the positive emotional polarity of the supporting services has a weak impact on consumer shopping decisions. This is mainly because JD.com’s logistics service has already formed a relatively mature system. Specifically, JD.com has established its own logistics network and after-sales service system, and also proposed a series of service measures such as “order today, arrive tomorrow,” “211 limited time delivery,” and “100 points after-sales” (Liang, 2019). Consumers have an overall perception of the efficiency of their supporting services. Therefore, the positive emotional polarity of online reviews about supporting services has a limited impact on the purchase decisions made by consumers.

(3) Influence of Negative emotional polarity of online reviews on consumers’ purchase decisions under different factors:

H3a and H3b predict the influence of the negative emotional polarity of the online review for the product quality factor (ln *QN*_*i*,*t*_) and the support service factor (ln *SN*_*i*,*t*_) on consumers’ purchase decisions. As can be seen from Table 4 Model 3, the negative emotional polarity of online reviews under both factors has an extremely significant negative impact on consumer purchase decisions (α_7_ = −10.017, *p* < 0.01; α_8_ = −0.659, *p* < 0.01). Being a newly launched product, it is more difficult for consumers to understand the features and supporting services of laptops. In this case, consumers are likely to be empathy-inspired and impressed by the problems mentioned in the reviews. And they are extremely prone to attribute negative reviews to external sources, that is, they may believe that it is the quality of the product and the logistics and after-sales services that caused the reviewer’s dissatisfaction (Sen and Lerman, 2007). Thus, such reviews with negative emotions tend to warn potential consumers to deter them from making a purchase decision.

(4) Influence of variance of online reviews on consumers’ purchase decisions under different factors:

According to Model 3 in Table 4, it can be seen that the online review variance of the support service factor (ln *SA*_*i*,*t*_) has a significant negative effect on consumer purchase decisions (α_10_ = −0.547, *p* < 0.05), supporting H4b. However, the online review variance of the product quality factor (ln *QA*_*i*,*t*_) does not have a significant effect on consumer purchase decisions and does not support H4a. This may be related to the subject of this paper, the new products. New products are more unique than existing established products because they make changes in the attributes of the product itself such as design, appearance, and concept (Kuijken et al., 2017). This unique characteristic of the new product will give rise to mixed opinions, which may, in some way, stimulate heated consumer debate (Chen and Berger, 2013). As a result, potential consumers will attribute the variance portion of the review to the uniqueness of the new product and research the review more closely rather than rejecting the purchase outright. Therefore, the online review variance of new product quality did not have a significant negative effect on consumer purchase decisions.

Judging from the estimated results of the control variables from Model 3, the negative effect of price on the purchase decision of new products is also in line with the laws of economics, that is, price is an important factor affecting sales, and consumer purchases will decline as prices rise. The effect of review timeliness on consumer purchase decisions was not significant. The possible reason is that new products have a relatively short time to market, most online reviews of products have a relatively short time interval, and therefore have a limited role in influencing consumer purchase decisions. A longer review length indicates that the review contains relatively more information about the product, which helps consumers to gain an indirect consumer experience about the new product and therefore has a stronger impact on consumer shopping decisions. Whereas promotions act as a valuable stimulus signal, regardless of country, age, income, education, or marital status, promotions of goods will make consumers feel good about the product and its quality (Fam et al., 2019), which will have a significant positive effect on consumer purchases.

We compare 2SLS (Model 3) with OLS (Model 1) and find some significant changes in the importance of the variables. In particular, ln *QA*_*i*,*t*_ is an important predictor in OLS estimation. We find that the 2SLS results about ln *QA*_*i*,*t*_ are in contrast to the results of OLS regression. The OLS results show that the online review variance of product quality has a significant negative effect on consumer purchase decisions. However, after controlling for endogeneity, this effect is no longer significant. This may provide a new explanation for the heterogeneity of impact studies in previous studies addressing the variance of online reviews. Considering the correlation between the error term and the endogenous variables, a simple OLS regression cannot properly describe the effect of online review variance. In the context of our particular study, the effect of ln *QA*_*i*,*t*_ is overestimated in OLS, considering the endogeneity of ln Volume_*i*,*t*_. We note that the coefficient of ln *Volume*_*i*,*t*_ increases from 0.611 in OLS to 0.662 in 2SLS, which is a noteworthy difference. This implies that not considering the endogeneity of ln *Volume*_*i*,*t*_ leads to an underestimation of its impact on consumer purchase decisions. Other significant differences in the coefficients include ln *QV*_*i*,*t*_ (0.572 in OLS to 0.596 in 2SLS), ln *SP*_*i*,*t*_ (0.229 in OLS to 0.093 in 2SLS) and ln *QN*_*i*,*t*_ (−0.877 in OLS to −1.017 in 2SLS) and ln *SN*_*i*,*t*_ (−0.621 in OLS to −0.659 in 2SLS).

### Robustness test based on simultaneous equations

In the econometric regression model, the presence of endogeneity problems will lead to biased and variance in the estimation results. Although, we have controlled for important variables that influence consumers’ purchase decisions and dealt with the endogeneity problem due to the possible omission of variables by adding instrumental variables using 2SLS. However, the possible bi-directional causality between the volume of online reviews and consumers’ decision to purchase a new product can also lead to endogeneity problems. That is, not only does the volume of online reviews affect consumers’ shopping decisions but also consumer purchases, as important market behavior, may have an impact on the volume of online reviews (Hu et al., 2009). Therefore, the one-equation estimation method may ignore this correlation, leading to endogeneity problems (Rothenberg and Leenders, 1964; Li and Liu, 2005). Therefore, we will refer to the practice of Li and Liu (2005), based on the single equation model, using the simultaneous equation model for further exploration.

We use the baseline model as the purchase decision equation in the set of simultaneous equation models, that is Eq. (5). Meanwhile, consideration of the emotional polarity (positive and negative) of online reviews and the variance of online reviews also affects the volume of online reviews (Wang et al., 2015; Wang and Guo, 2016); therefore, these three factors are also introduced into Eq. (6) for investigation. Therefore, the extended simultaneous equations are as follows:


(5) $$lnPurchase_{i,t}={\text{θ}}_{t}+{\text{α}}_{1}lnPurchase_{i,t-1}+{\text{α}}_{2}\ln Volume_{i,t}$$

$$\;+{\text{α}}_{3}\ln QV_{i,t}+{\text{α}}_{4}\ln SV_{i,t}++{\text{α}}_{5}\ln QP_{i,t}$$

$$\;+{\text{α}}_{6}\ln SP_{i,t}+{\text{α}}_{7}\ln QN_{i,t}+{\text{α}}_{8}\ln SN_{i,t}$$

$$+{\text{α}}_{9}\ln QA_{i,t}+{\text{α}}_{10}\ln SA_{i,t}$$

$$\;+\sum {\text{ρ}}^{*}{\mathrm{Control}}_{i,t,}+{\text{μ}}_{i}+{\text{ε}}_{i,t}$$

(6) $$\ln Volume_{i,t}={\text{η}}_{t}+{\text{γ}}_{1}lnPurchase_{i,t}+{\text{γ}}_{2}\ln Pemotional_{i,t}$$

$$\;+{\text{γ}}_{3}lnNemotional_{i,t}+{\text{γ}}_{4}\ln Variance_{i,t}$$

$$+\sum {\text{ρ}}^{*}{\mathrm{Control}}_{i,t}+{\text{β}}_{i}+{\text{σ}}_{i,t}$$

Where ln *Pemotional*_*i*,*t*_, *lnNemotional*_*i,t*_, and *lnVariance*_*i,t*_ are the positive emotional polarity of online reviews, the negative emotional polarity of online reviews, and the variance of online reviews, respectively. In addition, η_*t*_ is the intercept terms of the Eq. (6), respectively, and represents the total time difference of each newly launched laptop. σ_*i,t*_ represents the residual term. The definitions of other variables are the same as above. There are two endogenous variables in this simultaneous equation, namely, consumer purchasing decisions and the volume of online reviews, and other variables of the model are exogenous variables.

The three-stage squares estimation (3SLS) is an effective way to estimate the simultaneous equations. As a typical system estimation method, the 3SLS method is based on 2SLS estimation and uses the moment matrix of the perturbation terms of the structural equations obtained by 2SLS estimation to estimate the coefficients of the whole system equations simultaneously. Because 3SLS allows correlations between unobserved disturbances in the various equations to be used in the analysis, consistent estimates can be obtained (Khuda et al., 2017). Consumer purchasing decisions and online reviews are susceptible to a combination of variables. In this case, the 3SLS is also suitable. A prerequisite for parameter estimation with a simultaneous equation model is that its parameters must be “identifiable.” To identify the structural equation, the number of exogenous variables excluded by the structural equation should be greater than (over-identified) or equal to (exactly identified) the number of endogenous explanatory variables contained in the equation (Olusegun and Oyejola, 2015). According to the order condition and rank condition identified by the simultaneous equation model, the simultaneous equation model constructed in this paper is over-identified. On this basis, the 3SLS estimation is also suitable for situations where some variables in the simultaneous equations are endogenous. The volume of online reviews and purchase decisions are taken as two endogenous variables in the estimation strategy. For the selection of instrumental variables, we still choose the total number of useful votes with one lag used in the 2SLS estimation as the instrumental variable for the volume of online reviews and use the lagged term of the purchase decisions as its instrumental variable, thus forming the “just-identified” state of the instrumental variable equation, which is used to solve the endogeneity problem solved by He et al. (2020).

The 3SLS regression results are presented in the Table 5 and analyzed in comparison with the previous two-stage estimation results of 2SLS (Model 3). The Table 5 shows that although there are differences in the degree of influence of each explanatory variable on consumer purchase decisions, the sign of the statistical coefficients remains unchanged, indicating that the baseline regression results are robust and reliable. After considering the endogeneity problem due to causality, the facilitation effect of the volume of online reviews on consumer purchase decisions significantly increased, from 0.662 to 0.697 with the same level of significance, which indicates that the effect of the impact of the volume of online reviews on the consumer purchase decision is shifted upward by controlling for endogeneity through the 3SLS and further validates the facilitation effect. Also, for the 3SLS estimation results of review Eq. 5, the consumer purchase decision significantly and positively affects the volume of online reviews, that is, the higher the degree of consumers buying new products, the more online reviews will increase accordingly. Combined with H1, it illustrates that online reviews of new products not only directly influence consumers’ purchase decisions, but are also reactors of purchase decisions. Online reviews play a dual role in consumer purchase decisions. Also, although there are some differences in the magnitude and significance of the coefficients on the role of the volume of online reviews of product quality and supporting services, positive and negative emotional polarity, and variance on the purchase decision, whether they are significant and the direction of the effect is consistent with the 2SLS estimates. Regarding the results of the remaining control variables, only the significance of the effect of promotional variables on consumer purchase decisions for new products differed somewhat. Therefore, the regression results in this paper are generally robust. This indicates that overall, online reviews have a more significant effect on consumers’ purchase decisions, but the effect of online reviews on consumers’ purchase decisions varies across product characteristics.

**TABLE 5:** Regression results of simultaneous equations.

	Simultaneous equation system	
	Purchase decisions equation	Online review equation
*lnPurchase*_*i,t*_	–	0.671*** (0.119)
*lnPurchase*_*i,t–1*_	0.792*** (0.233)	–
ln *Volume*_*i, t*_	0.697*** (0.136)	–
ln *QV*_*i*,*t*_	0.683*** (0.096)	–
ln *SV*_*i*,*t*_	0.521** (0.209)	–
ln *Pemotional*_*i, t*_	–	0.319 (0.213)
ln *QP*_*i*,*t*_	0.381* (0.125)	–
ln *SP*_*i*,*t*_	0.299* (0.109)	–
ln *Nemotional*_*i, t*_	–	0.530** (0.199)
ln *QN*_*i*,*t*_	−0.886*** (0.286)	–
ln *SN*_*i*,*t*_	−0.772* (0.349)	–
ln *Cuality*_*i, t*_	–	0.247* (0.124)
ln *QA*_*i*,*t*_	−0.431 (0.200)	–
ln *SA*_*i*,*t*_	0.339* (0.195)	–
*lnPrice*_*i,t*_	−0.209* (0.113)	0.633** (0.212)
*lntimeliness*_*i, t*_	0.039 (0.128)	0.280 (0.207)
*lnLenth*_*i,t*_	0.236* (0.124)	0.391* (0.216)
*Spromotion*_*i,t*_	0.417 (0.269)	0.237 (0.258)
Cons-	3.138** (1.211)	3.673** (1.395)
N	45801	45801
R^2^	0.733	0.697

*p < 0.05, **p < 0.01, ***p < 0.001; values in parentheses are robust standard errors.

### Analysis of interaction effect results

Based on the benchmark model, we will apply the 2SLS method to verify the interaction effects among online review metrics under the product quality factors. Before conducting the regressions, the data were centralized by subtracting the means of the respective variables to minimize the effect of multicollinearity problems between the explanatory and control variables on the test results. The sample mean of the treated variables is zero and the sample distribution is the same as before the treatment. Then, the multicollinearity diagnosis indicated that all VIF values were less than 5, which indicated that the multicollinearity problem was not serious and did not pose an impact on the test results. Based on this, we put the interaction terms of the volume and positive emotional polarity of online reviews, the volume and negative emotional polarity of online reviews, the variance and volume of online reviews, the positive emotional polarity and variance of online reviews, and the negative emotional polarity and variance of online reviews of product quality into Models I, II, III, IV, and V separately, in that order. The coefficients and significance of the other explanatory variables did not change significantly during the joining process, and the overall regression was robust.

First, the regression results of Model I in Table 6 show that the interaction between online review volume and positive emotional polarity (ln QV_i,t_* ln *QP_i,t_*) and consumer purchase decisions have insignificant effects. This may be because a large number of online reviews with positive sentiments may lead potential consumers to question the authenticity of the reviews. This phenomenon stems from the unethical behavior of online sellers in recent years, who tend to post positive and false reviews of their products to gain financial benefits (Wang et al., 2018). This has led consumers to worry about collusive fraudulent transactions in e-commerce when faced with a large number of positive online reviews (Saia et al., 2019), and they believe that stores may have hired specialized personnel to write fake reviews. As a result, consumers no longer experience significant mood swings when confronted with these online reviews. As demonstrated by Doh and Hwang (2009) in an online review balancing experiment, when the proportion of positive reviews on a website is too high, it raises consumer suspicion.

**TABLE 6:** Regression of interaction effects estimated by two-stage least squares method (2SLS).

	Model I	Model II	Model III	Model IV	Model V
**Explanatory variables**					
*lnSales*_*i,t–1*_	0.557** (0.079)	0.737*** (0.141)	0.103*** (0.11)	0.686*** (0.166)	0.781*** (0.159)
ln *Volume*_*i*, *t*−1_	0.68*** (0.034)	0.568*** (0.085)	0.701*** (0.062)	0.534*** (0.108)	0.654*** (0.117)
ln *QV*_*i*,*t*−1_	0.624** (0.192)	0.625*** (0.081)	0.822** (0.125)	0.884*** (0.176)	1.004*** (0.191)
ln *SV*_*i*,*t*−1_	0.513** (0.098)	0.541** (0.175)	0.539** (0.192)	0.518** (0.131)	0.566* (0.15)
ln *QP*_*i*,*t*−1_	0.253* (0.152)	0.301** (0.145)	0.390** (0.125)	0.312** (0.129)	0.334* (0.129)
ln *SP*_*i*,*t*−1_	0.344 (0.4)	0.317 (0.437)	0.380 (0.397)	0.291 (0.414)	0.299 (0.443)
ln *QN*_*i*,*t*−1_	−0.74*** (0.151)	−0.723** (0.290)	−0.694*** (0.206)	−0.73*** (0.219)	−0.718*** (0.217)
ln *SN*_*i*,*t*−1_	−0.364* (0.103)	−0.385* (0.142)	−0.477* (0.124)	−0.351 (0.185)	−0.39* (0.116)
ln *QA*_*i*,*t*−1_	−0.776 (0.629)	−0.752 (0.662)	−0.545 (0.657)	−0.692 (0.628)	−0.758 (0.657)
ln *SA*_*i*,*t*−1_	−0.445* (0.124)	−0.433* (0.164)	−0.705* (0.157)	−0.424* (0.177)	−0.498* (0.178)
**Interaction terms**					
ln *QV*_*i*,*t*−1_*ln *QP*_*i*,*t*−1_	0.590 (0.371)	–	–	–	–
ln *QV*_*i*,*t*−1_*ln *QN*_*i*,*t*−1_	–	−0.672* (0.349)	–	–	–
ln *QA*_*i*,*t*−1_*ln *QV*_*i*,*t*−1_	–	–	0.471** (0.104)	–	–
ln *QP*_*i*,*t*−1*_ln *QA*_*i*,*t*−1_	–	–	–	−0.312* (0.138)	–
ln *QN*_*i*,*t*−1_*lnQA_*i*,*t*−1_	–	–	–	–	−0.361 (0.437)
**Control variables**					
*ln*Pric*e*_*i*,*t*_	−0.015** (0.033)	−0.01** (0.006)	−0.008** (0.028)	−0.006** (0.026)	−0.007** (0.035)
*ln*timeliness_*i*, *t*_	0.386* (0.135)	0.379* (0.162)	0.413 (0.319)	0.382** (0.194)	0.362* (0.179)
*lnLenth*_*i,t*_	0.274* (0.167)	0.462* (0.190)	0.412 (3.239)	0.394* (0.151)	0.259* (0.132)
*Spromotion*_*i,t*_	0.459 (0.213)	0.426* (0.105)	0.419 (0.281)	0.412* (0.163)	0.411 (0.306)
Cons-	3.613* (1.427)	3.614* (1.427)	3.618* (1.432)	3.614* (1.429)	3.443* (1.413)
N	45801	45801	45801	45801	45801
R^2^	0.747	0.748	0.753	0.747	0.748

*p < 0.05, **p < 0.01, ***p < 0.001; values in parentheses are robust standard errors.

Second, the regression results of Model II in Table 6 show that the relationship between online review volume and online review negative emotional polarity (ln QV_i,t_* ln *QN_i,t_*) and consumer purchase decision is significant (β_2_ = −0.672, *p* < 0.05), which means that the interaction between the volume of online reviews and the negative emotional polarity of online reviews will discourage consumers from purchasing new products. The negative emotional polarity of online reviews implies that consumers are negative and critical of the product experience, thus, when faced with negative reviews of laptops, the increase in the volume of reviews will undoubtedly reinforce the perception that the product is defective, thus preventing consumers from making a purchase decision.

Then, the regression of Model III in Table 6 shows that the interaction terms of online review variance and online review volume (ln QA_i,t_* ln *QV_i,t_*) are positively related to consumer purchase decision (β_3_ = 0.471, *p* < 0.01). This suggests that the volume of online reviews attenuates the negative effect of online review variance on consumer purchase decisions. And, information overload due to high variance and the high number of online reviews can trigger heuristics that allow consumers to focus on the uniqueness of a new product, thereby enhancing the consumer’s purchase decision. The results again support the importance of the number of online reviews as a high-range cue for consumers’ purchase decisions (Park and Jang, 2013). Our findings also validate the point made by Khare et al. (2011) that a higher volume of online reviews is more diagnostic and absorbs negative consumer perceptions of online review goals, relative to a low online review volume.

Finally, the regression results of Models IV and V in Table 6 show that the increase in the variance of online reviews of new products attenuates the positive effect of positive emotional polarity of online reviews (ln QP_i,t_* ln *QA_i,t_*) on consumer purchase decisions (β_*4*_ = −0.312, *p* < 0.05), supporting H5d. However, the interaction between negative emotional polarity and variance of online reviews (ln QN_i,t_*lnQA_i,t_) has no significant effect on consumer purchase decisions, so H5e is not supported. This result is not fully consistent with the conclusion proposed by Kostyra et al. (2016) that uncertainty, represented by high variance, is favorable for products with low ratings and unfavorable for products with high ratings. We argue that consumer preference for positive online reviews is lower when the variance of online reviews is higher, but has a limited role in mitigating consumer resistance to negative online reviews. This may be since consumers are more sensitive to the perceived risks of new products due to the lack of calibration of product expectations (Xiao et al., 2018). Faced with negatively emotionally polarity online reviews, even though they see that some reviews have made positive comments about the product, they still tend to reduce the risk by simply not buying the product for safety reasons (Yan et al., 2018).

## Conclusion

Our research contributes to theory and practice. By applying the LDA topic model to identify the topics consumers pay attention to, we provided a theoretical framework to understand the impact of online reviews on purchase decisions. Specifically, we provided a multi-dimensional research framework to illustrate that, under different new product attribute dimensions (product quality or supporting services), the volume, the emotional polarity, and variance of new product online reviews have different effects on a purchase decision. Meanwhile, we also found that the interaction between the metrics of online reviews also has a complex impact on the purchase decision. In particular, the interaction between the volume of online reviews and positive emotional polarity did not facilitate consumer purchase decisions as much as we expected.

### Theoretical implications

Several theoretical implications may be derived from our study. First, this study contributes to academic research with a new data source and research approach for studying the influence of online reviews on consumer purchase decisions. Unlike traditional questionnaires and field interviews used in previous studies, we used online reviews as the primary data source. And based on a large number of review contents, we used the LDA model to identify the most concerned factors of consumers, including the appearance design factor, laptop setup factor, logistics service factor, after-sales service factor, and user experience factor; and then divide these factors into two categories: product quality factor and supporting service factor. What is more, our study shows that there are significant differences in the influence of online reviews on consumers’ purchase decisions under the product quality factor and the supporting services factor. In particular, high variance in online reviews of supporting services can discourage consumers from purchasing new products. In contrast, the variance of online reviews under the product quality factor does not have an impact on consumers’ purchase decisions because of the qualities that distinguish new products from established products. This finding highlights the importance of distinguishing the aspects of different product attributes when studying the impact of online reviews, and enriches previous research on the impact of online reviews.

Then, we also explored the impact of interactions between metrics of online reviews on the play of consumer purchase decisions, extending the study of the impact of separate metrics of online reviews. We found an interesting phenomenon that the interaction between the volume of online reviews and positive emotional polarity does not affect consumers’ purchase decisions. That is, consumers do not follow the positive guidance and thus make a purchase decision when faced with a large number of positive emotional polarities. This is in line with the current state of development of China’s e-commerce environment. In recent years, with the development of e-commerce platforms, many merchants have hired people to write fake reviews to make consumers believe that the product is superior (Jiménez and Mendoza, 2013). Increasingly, consumers are becoming capable of identifying false positive reviews in product reviews and will no longer experience strong emotional swings. This finding advances the research of Floyd et al. (2014), who mainly conducted a meta-analysis on the impact of the interaction between valence and volume on sales. Our study provided new insights into exactly how positive emotional polarity affects the consumer purchase decision.

Finally, we also found that the interaction between online review variance and negative affective polarity of online reviews did not contribute to consumer purchase decisions. This suggests that consumers are extremely sensitive to information with negative affective polarity. In order to avoid losses, they would quickly decide not to purchase when confronted with negative affective polarity online reviews, regardless of the heterogeneity of the reviews. This means that consumers are less engaged in processing information when they receive negative emotional polarity reviews than when they receive positive emotional polarity reviews. This finding develops the gatekeeping role of review efficacy implied by the loss aversion theory proposed by Jia and Liu (2018).

### Managerial implications

First, companies should pay attention to the importance of online reviews for the purchase decision, especially the text mining of online review content. As discussed in the literature, online reviews play an important role as heuristic attributes for consumers to generate purchase intentions and make purchase decisions (Lovett et al., 2013). Therefore, companies should make full use of online reviews as a new form of digital marketing to stimulate consumers to buy new products. They can invest more resources in online review systems, and monitor and respond to online reviews. In addition, through the text mining based on the LDA model, we found that the product quality and supporting services of laptops are the focus of consumers. Therefore, companies can introduce incentive programs to encourage consumers to provide feedback in the form of structured online reviews; and encourage consumers to mention details about product quality and service as much as possible, especially to deliver real experience about product quality to potential users. Therefore, the appeal of online reviews to consumers can be increased by using a structured online review format. Companies and marketers can combine online reviews with text mining and analysis, and quantify unstructured data, thereby obtaining consumer demand preferences and product information that is more conducive to making purchasing decisions. We argued that text mining and analysis for online review content allows marketers to quantify unstructured data as a way to help companies obtain leads from specific review content that are more likely to motivate consumers to purchase new products.

Meanwhile, companies and marketers should encourage consumers to actively review and share their purchase and use experiences on time, and to awaken their awareness of new products by providing potential consumers with more information about them, thus stimulating their willingness to buy. However, companies do not need to guide reviewers to make positive emotional reviews but should encourage their customers to provide more fair and honest reviews. This is because when there are a large number of positive reviews, potential consumers do not follow this positive guidance and make a decision to buy a new product. Moreover, the negative affective polarity of online reviews can negatively influence consumers’ purchasing decisions, especially concerning negative reviews about product quality. Therefore, the most fundamental thing for companies is to try to make good products to prevent negative reviews from the source.

In addition, corporate marketers should monitor and analyze online data and pay attention to negative reviews posted by consumers promptly. Companies should not be overly afraid of negative sentiment polarity reviews once they occur, nor should they hide negative reviews through intervention. A consumer survey by Weisstein et al. (2017) shows that when online shoppers see online sellers’ reactions to negative reviews, their purchase intentions will double, instead of just focusing on negative reviews. Therefore, what companies need to do when facing negative emotional polarity reviews is to quickly analyze customers’ pain points and shortcomings in terms of products and services, promptly handle complaints from consumers and strive to minimize the impact of negative emotional polarity online reviews as a way to ensure that potential consumers can purchase new products.

### Limitations and future research

Although this paper systematically studies the influencing factors and their mechanism for consumer purchase decisions based on online reviews, due to various limitations, this paper still has some shortcomings and needs to be improved. First, the online review text information selected in this paper only comes from laptop products. In future research, we will add other types of products as the research object to verify the applicability of our research methods. Second, this paper only uses the JD platform as the data source. In future research, we will consider adding e-commerce platforms such as Tmall and Suning.com. Through comparative analysis, we will further explore the law of consumer purchase decisions from the perspective of online reviews. Finally, Although the LDA topic model has more advantages, the LDA model is computationally very expensive on large datasets. Therefore, a more effective methods will be needed to face the large number of online review researches on multiple platforms. In the future, we will try to explore the utility of deep learning and topic modeling algorithms in online consumer reviews. Specifically, deep learning models such as Word-to-Vector and Sentence-to-Vector (Bastani et al., 2019) can be used for continuous representation of documents and embedding them in much lower dimensions while better capturing the semantic similarity of documents.

## Data availability statement

The raw data supporting the conclusions of this article will be made available by the authors, without undue reservation.

## Author contributions

MK was mainly responsible for the proposal of the research topic of the thesis, the construction of the model, and the invocation of the software. BS was mainly responsible for the correction of the thesis selection, the writing of the thesis, and the correction of the problems at the later stage of the thesis. TL was mainly responsible for the collection and organization of the data and the writing of the thesis. H-YM was mainly responsible for the collection and organization of the thesis data and the correction at the later stage of the thesis. All authors contributed to the article and approved the submitted version.
